# SPI-1-encoded type III secretion system of *Salmonella enterica *is required for the suppression of porcine alveolar macrophage cytokine expression

**DOI:** 10.1186/1297-9716-42-16

**Published:** 2011-01-24

**Authors:** Barbora Pavlova, Jiri Volf, Petra Ondrackova, Jan Matiasovic, Hana Stepanova, Magdalena Crhanova, Daniela Karasova, Martin Faldyna, Ivan Rychlik

**Affiliations:** 1 Veterinary Research Institute, Brno, Czech Republic

## Abstract

Genes localized at *Salmonella *pathogenicity island-1 (SPI-1) are involved in *Salmonella enterica *invasion of host non-professional phagocytes. Interestingly, in macrophages, SPI-1-encoded proteins, in addition to invasion, induce cell death via activation of caspase-1 which also cleaves proIL-1β and proIL-18, precursors of 2 proinflammatory cytokines. In this study we were therefore interested in whether SPI-1-encoded type III secretion system (T3SS) may influence proinflammatory response of macrophages. To test this hypothesis, we infected primary porcine alveolar macrophages with wild-type *S*. Typhimurium and *S*. Enteritidis and their isogenic SPI-1 deletion mutants. ΔSPI1 mutants of both serovars invaded approx. 5 times less efficiently than the wild-type strains and despite this, macrophages responded to the infection with ΔSPI1 mutants by increased expression of proinflammatory cytokines IL-1β, IL-8, TNFα, IL-23α and GM-CSF. Identical macrophage responses to that induced by the ΔSPI1 mutants were also observed to the infection with *sipB *but not the *sipA *mutant. The *hilA *mutant exhibited an intermediate phenotype between the ΔSPI1 mutant and the wild-type *S*. Enteritidis. Our results showed that the SPI-1-encoded T3SS is required not only for cell invasion but in macrophages also for the suppression of early proinflammatory cytokine expression.

## Introduction

*Salmonella enterica *is an obligate pathogen which can successfully colonize different hosts including reptiles, birds and mammals. Out of many genes which contribute to its virulence, two of the most important genetic loci are those coding for two different type III secretion systems (T3SS) localized at *Salmonella *pathogenicity islands 1 and 2 (SPI-1 and SPI-2). The SPI-2-encoded T3SS enables *S*. *enterica *to survive and proliferate in phagolysosomes inside different cell types, macrophages in particular [1,2] while the SPI-1-encoded T3SS enables *S. enterica *invasion into non-professional phagocytes [2]. However, on a level of animal-pathogen interaction, SPI-1 mutants can be found easily in mesenterial lymph nodes, liver or spleen in mice, chickens or pigs [2-5] which indicates that in vivo, the invasion of epithelial cells is not strictly required for *S. enterica *pathogenesis and the SPI-1-encoded T3SS may have additional function(s) during the in vivo infection, as proposed earlier [2].

After oral ingestion and multiplication in the gut lumen, *S*. *enterica *translocates across the gut epithelium into mucosa where it comes into contact with resident macrophages. The interaction of *S. enterica *with macrophages can result in two mutually exclusive events - *S. enterica *can either survive inside macrophages and can use them for spreading throughout the host's body, or it can be cytotoxic for macrophages and induce early macrophage cell death. How the balance between the two alternatives is regulated is not known. However it has been reported that the cytotoxicity induced by *Salmonella *is dependent on the SPI-1-encoded SipB protein and host's caspase-1 [6] and that this occurs quite specifically in macrophages and not in other cell types [7,8]. Besides the other functions, activated caspase-1 then cleaves proIL-1β and proIL-18 into their biologically active forms that initiate inflammation [9].

Because of the above mentioned facts, we sought alternative function(s) of SPI-1 in *S. enterica *pathogenesis and one of the hypotheses which we considered was that the proteolytic activation of IL-1β and IL-18 may lead to an increased transcription of other proinflammatory cytokines. If this hypothesis was correct, it could be possible to detect changes in mRNA levels of additional proinflammatory cytokines and other factors by RT PCR in macrophage populations infected with *S. enterica*. We therefore exposed porcine alveolar macrophages (PAMs) to the wild-type *S. enterica *serovars Typhimurium and Enteritidis (*S*. Typhimurium and *S*. Enteritidis) and their isogenic ΔSPI1 mutants and quantified the transcription of 14 cytokine or immune response relevant genes. Unlike all previous reports, we observed that the absence of SPI-1 resulted in a higher cytokine expression of PAMs when compared with the PAMs exposed to the wild-type strains. This showed that besides cell invasion, an additional function of SPI-1-encoded T3SS is the suppression of cytokine expression in infected macrophages and therefore the suppression of proper immune response of a host.

## Materials and methods

### Bacterial strains and growth conditions

*S*. Enteritidis 147 [10] and *S*. Typhimurium 10C7 belonging to phage-type DT104, and their isogenic ΔSPI1 mutants were used in the study. Construction of *S*. Enteritidis ΔSPI1 mutant has been described previously [5] and *S*. Typhimurium ΔSPI1 mutant was constructed newly in this study in exactly the same way. In *S*. Enteritidis, single gene mutants in *hilA*, *sipB *and *sipA *genes localized at SPI-1, characterized previously [11], were used in some of the experiments. Before the macrophage infection, overnight cultures were diluted 500 × in LB broth and incubated for 6 h at 37 °C to obtain cultures with maximally expressed SPI-1 genes [7].

### Isolation and cultivation of PAMs

PAMs were obtained from the lungs of clinical healthy pigs immediately after slaughter by bronchoalveolar lavage as described previously [12]. Isolated PAMs (approx. 2 × 10^5 ^of PAMs per well of 24-well microplates) were allowed to attach for at least 1 h in DMEM medium (Gibco, Carlsbad, USA) containing antibiotics (gentamicin 4 μg/mL; penicillin 100 U/mL; streptomycin 100 μg/mL). After that, porcine serum (Gibco, USA) was added to 10%. After 16-h cultivation, just prior to the infection with *Salmonella*, the medium was replaced with DMEM supplemented with 10% of porcine serum and free of any antibiotics.

*Salmonella *was added to PAMs in a multiplicity of infection (MOI) equal to 5 and the *Salmonella*-PAM interaction was terminated either 4 or 24 h later. One hour after the infection, 100 μg/mL of gentamicin was added to kill any extracellular *Salmonella*. After another hour, the medium with 100 μg/mL of gentamicin was replaced with fresh medium containing 15 μg/mL of gentamicin to prevent extracellular *Salmonella *replication. In parallel, the numbers of intracellular *Salmonella *in PAMs were determined 4 h and 24 h after the infection by lysis with 1% Triton X-100 for 20 min. The suspensions were then serially diluted and the dilutions were plated on LB agar. Viability of PAMs was monitored based on the release of lactate dehydrogenase (LDH) into culture medium using CytoTox 96^® ^Non-Radioactive Assay according to the instruction of the manufacturer (Promega, Madison, USA). LPS (1 μg/mL) was used as a positive control of macrophage stimulation. Negative controls included assay performed with tissue culture medium or PAMs without any contact with *Salmonella*.

The experiments were performed on three independent occasions - in the first set of infections we obtained PAMs from 6 pigs and infected them with *S*. Typhimurium and its ΔSPI1 isogenic mutant. In the second experiment we obtained PAMs from four pigs and infected them with *S*. Enteritidis and its ΔSPI1 isogenic mutant. In the third experiment we obtained PAMs from six pigs and infected them with *S*. Enteritidis ΔSPI1 and single gene *hilA*, *sipB *and *sipA *mutants.

### Cytokine gene expression determined by RT PCR and ELISA

Total RNA was purified using the RNeasy Mini Kit according to the manufacturer's instructions (Qiagen, Hilden, Germany). The quantity and quality of RNA was checked spectrophotometrically and by agarose gel electrophoresis. Purified RNA was reverse transcribed with 200 U of M-MLV reverse transcriptase (Invitrogen, Carlsbad, USA) and oligo-dT primers, and the cDNA was used either immediately or was stored at - 20 °C until use. Genes targeted in RT-PCR included GM-CSF, IL-1β, IL-6, IL-8, TNFα, IL-12α, IL-12β, IL-18, IL-23α, TGFβ, iNOS, p47^phox^, SOD1 and Nramp1 (Slc11a1). Primers for GM-CSF, TNFα and HPRT were adopted from [13], IL-8 primers were designed according to [14] and primers for iNOS were adopted from [12]. The remaining primers were designed using Primer3 software and all the primers are listed in Table 1. RT-PCR was performed with QuantiTect SYBR Green PCR Kit (Qiagen) using the LightCycler 480 (Roche, Basel, Switzerland). HPRT mRNA was used as a house keeping reference gene and the threshold cycle values (C_t_) of gene of interest were first normalized to the C_t _value of HPRT reference mRNA (ΔC_t_) and the normalized mRNA levels were calculated as 2^(-ΔC^_t_^)^. The normalized mRNA levels of a particular cytokine ± standard deviation are shown in the figures as "HPRT units".

**Table 1:** List of primers used for the RT PCR quantification of gene expression

	Sequence 5'-3'	Reference
IL1βFor	GGGACTTGAAGAGAGAAGTGG	this study
IL1βRev	CTTTCCCTTGATCCCTAAGGT	this study
IL8For	TTCTGCAGCTCTCTGTGAGGC	[14]
IL8Rev	GGTGGAAAGGTGTGGAATGC	[14]
TNFβFor	CCCCCAGAAGGAAGAGTTTC	[13]
TNFβRev	CGGGCTTATCTGAGGTTTGA	[13]
IL23p19αFor	GACAACAGTCAGTCCTGCTTGC	this study
IL23p19αRev	ACAGAGCCATCAGGGTGTAGAGA	this study
IL12p40βFor	CACTCCTGCTGCTTCACAAA	this study
IL12p40βRev	CGTCCGGAGTAATTCTTTGC	this study
IL12p35αFor	AACTAGCCACGAATGAGAGTTGC	this study
IL12p35αRev	GCACAGGGTTGTCATAAAAGAGG	this study
TGFβFor	TACGCCAAGGAGGTCACCC	this study
TGFβRev	CAGCTCTGCCCGAGAGAGC	this study
IL18For	ATGCCTGATTCTGACTGTTC	this study
IL18Rev	CTGCACAGAGATGGTTACTGC	this study
GMCSFFor	TCTGTTGGCCAAGCACTATG	this study
GMCSFRev	GCAGTCAAAGGGGATGGTAA	this study
IL6For	CACCGGTCTTGTGGAGTTTC	this study
IL6Rev	GTGGTGGCTTTGTCTGGATT	this study
NRAMPFor	ATCAACCTCTTTGTCATGGCTGT	this study
NRAMPRev	AGATCTTGGCGTAGTCGTGGAG	this study
SOD1For	CATTAAAGGACTGGCTGAAGGTG	this study
SOD1Rev	CTGCACTGGTACAGCCTTGTG	this study
phox 47For	GACCATCGAGGTCATTCATAAGC	this study
phox 47Rev	ACATGGATGGGAAGTAACCTGTG	this study
iNOSFor	GTGATGGCCGACCTGATGTT	[12]
iNOSRev	GGCCCAGGAAATGTTCGAG	[12]
HPRTFor	GAGCTACTGTAATGACCAGTCAACG	[13]
HPRTRev	CCAGTGTCAATTATATCTTCAACAATCAA	[13]

Expression of IL-1β, IL-8 and TNFα was also determined in cell culture medium after 24-hour interaction with *S*. Typhimurium by ELISA according to the manufacturer's instructions (R&D System, Minneapolis, USA).

### Statistical analysis

Statistical analysis of differences in cytotoxicity and gene expressions induced by the wild-type strains and all the mutants was performed by paired *t*-test (Prism, Graph Pad Software, La Jolla, USA). Significant differences were defined as those with *p *< 0.05. In the case of cytokine expression, for the statistical analysis we used the ΔC_t _values since after the calculation of power, the deviations increased exponentially and many of the comparisons came out as insignificant with *p *values ranging between 0.05 - 0.10.

## Results

### Role of SPI-1 in the interaction of Salmonella with PAMs

**Figure 1:**
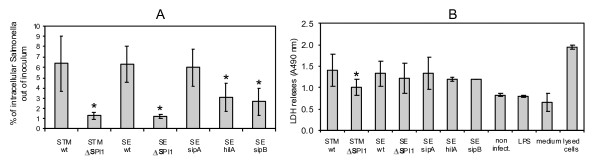
**Invasion and cytotoxicity of *S*. Typhimurium and *S*. Enteritidis for porcine alveolar macrophages**. Panel A, invasion of the wild-type *S*. Typhimurium (wt) and isogenic ΔSPI1 mutant, and *S*. Enteritidis (wt) and isogenic ΔSPI1, *sipA*, *hilA *and *sipB *mutants into porcine alveolar macrophages 4 h post infection. Panel B, cytotoxicity of *S*. Typhimurium, *S*. Enteritidis and their isogenic mutants for porcine alveolar macrophage 24 h post infection. Controls included LDH released from non-infected PAMs, PAMS exposed to LPS, tissue culture medium, and PAMs lysed with 1% Triton X-100. * significantly different at *p *< 0.05 when *t*-test compared with appropriate wild-type strain.

When the invasion, intracellular survival and cytotoxicity of *S*. Typhimurium, *S*. Enteritidis, and their ΔSPI1 mutants were tested, the ΔSPI1 mutants, independent of serovar, invaded significantly less efficiently than the wild-type strains (Figure 1A) but they survived inside macrophages as efficiently as the wild-type strains (not shown). When compared with the wild-type strain, the ΔSPI1 mutant of *S*. Typhimurium was significantly less cytotoxic for the porcine macrophages as measured by LDH release 24 h post addition of *S*. Typhimurium to macrophages. In *S*. Enteritidis, we did not observe any difference in the cytotoxicity between the wild-type strain and ΔSPI1 mutant for PAMs (Figure 1B).

### Cytokine response of PAMs to Salmonella SPI mutants

Next we determined the transcriptional response of PAMs infected with the wild-type *S*. Typhimurium and the ΔSPI1 mutant. Based on the expression profile, the tested genes could be clustered into four groups. First, expression level of IL-12α was below the detection limit of RT PCR (data not shown). The second group comprised iNOS, phox47, SOD1, Nramp1 and TGFβ in which a minimal transcriptional response to *S*. Typhimurium infection could be observed. The third group was formed by IL-6, IL-18 and IL-12β in which there was significantly increased expression in PAMs after contact with wild-type *S*. Typhimurium or the ΔSPI1 mutant but not all the differences at both exposure times were statistically significant. For the last group of cytokines, i.e. GM-CSF, IL-1β, IL-8, TNFα and IL-23α, the exposure of PAMs to wild type *S*. Typhimurium significantly increased their transcription and transcription of these cytokines was further significantly increased in PAMs infected with the ΔSPI1 mutant, both 4 and 24 h post infection (Table 2).

**Table 2:** Expression of cytokine or immune relevant genes in non-infected PAMs, and in PAMs after 4 and 24 h exposure to LPS (1 μg/mL), wild-type *S*. Typhimurium and ΔSPI1 mutant.

	4 hours				24 hours			
	**wt**	**ΔSPI1**	**LPS**	**non-infected**	**wt**	**ΔSPI1**	**LPS**	**non-infected**

IL-1β	153 ± 99.8	529 ± 319**	276 ± 104	2.05 ± 1.75**	19.5 ± 12.1	114 ± 83.8**	34.5 ± 25.7	0.16 ± 0.081**
IL-8	91.4 ± 76.2	232 ± 198**	179 ± 57.9	4.79 ± 1.68**	34.2 ± 27.9	140 ± 109**	62.4 ± 24.3	1.32 ± 1.17**
TNFα	79.8 ± 31.4	310 ± 116**	40.5 ± 9.25	2.19 ± 1.20**	3.33 ± 1.27	5.21 ± 2.14*	1.10 ± 0.24	0.33 ± 0.18**
IL-23α	22.0 ± 16.5	123 ± 87.4**	32.7 ± 15.5	0.24 ± 0.21**	9.26 ± 2.89	61.0 ± 22.9**	11.6 ± 6.24	0.066 ± 0.029**
GM-CSF	0.62 ± 0.56	2.63 ± 2.72**	0.97 ± 0.49	0.045 ± 0.018*	1.50 ± 0.53	5.14 ± 5.40**	1.61 ± 1.19	0.013 ± 0.0039*
IL-12β	0.15 ± 0.17	0.19 ± 0.20	0.39 ± 0.29	0.052 ± 0.048	0.32 ± 0.22	0.75 ± 0.46*	0.060 ± 0.046	0.0031 ± 0.0019**
IL-18	0.40 ± 0.25	0.51 ± 0.31*	0.54 ± 0.13	0.16 ± 0.041*	0.47 ± 0.15	0.68 ± 0.35	0.95 ± 0.47	0.16 ± 0.041**
IL-6	0.11 ± 0.13	0.35 ± 0.42**	0.21 ± 0.21	0.0027 ± 0.0028**	0.11 ± 0.16	0.11 ± 0.17	0.094 ± 0.092	0.0034 ± 0.0041**
TGFβ	32.0 ± 17.4	34.2 ± 18.6	37.1 ± 9.53	26.7 ± 8.57	42.1 ± 8.29	34.8 ± 7.04	25.5 ± 4.86	20.6 ± 10.1**
NRAMP1	60.7 ± 17.0	61.7 ± 13.8	47.9 ± 8.72	64.3 ± 14.9	85.7 ± 43.4	92.2 ± 37.1	46.9 ± 1.91	43.6 ± 9.13*
SOD1	14.8 ± 4.79	15.7 ± 2.29	18.4 ± 6.38	15.8 ± 2.70	14.7 ± 6.30	11.5 ± 2.21	11.5 ± 1.48	9.63 ± 1.89
phox47	5.08 ± 3.25	7.05 ± 3.98	3.44 ± 1.87	7.98 ± 3.70	3.52 ± 2.57	4.22 ± 3.30	2.44 ± 1.07	4.40 ± 1.98
iNOS	0.019 ± 0.015	0.051 ± 0.037*	0.040 ± 0.014	0.0035 ± 0.0029	0.0030 ± 0.0018	0.0026 ± 0.0016	0.0027 ± 0.0018	0.0031 ± 0.0025

* *p *< 0.05, ** *p *< 0.01 when compared to the PAMs infected with the wild-type strain

**Figure 2:**
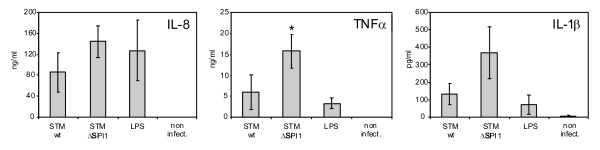
**ELISA detection of IL-1β, IL-8 and TNFα in tissue culture supernatants of non-stimulated PAMs and those exposed for 24 h to S. Typhimurium, ΔSPI1 mutant and LPS (1 μg/mL)**. *significantly different at *p *< 0.05 when *t*-test compared with the wild-type strain.

In the next experiment we verified the expression of IL-1β, IL-8 and TNFα by ELISA (Figure 2). Twenty-four hours post infection, all three cytokines were present in numerically higher amounts in PAMs exposed to the ΔSPI1 mutant than in those exposed to the wild type *S*. Typhimurium. However, the difference was statistically significant only for TNFα. For IL-1β and IL-8, the *p *values after the comparison of the wild type strain and ΔSPI1 mutant induction were 0.06 and 0.10, respectively. Since this experiment was performed in only three independent batches of PAMs originating from outbred pigs considerably affecting any statistical analysis and because the ΔSPI1 mutant in each of the batches always induced higher expression of each of the cytokines than did the wild type strain, we considered these results as consistent with the expression profiles determined by RT-PCR.

To determine whether this was really a SPI-1-dependent phenomenon independent of strain or serovar, we repeated the same experiment with exactly the same ΔSPI1 mutant constructed in *S*. Enteritidis. The results obtained with *S*. Enteritidis were similar to those observed in *S*. Typhimurium - macrophages responded by a significantly higher transcription of GM-CSF, IL-1β, TNFα, IL-23α and IL-12β to the infection with the wild-type *S*. Enteritidis when compared with the non-infected PAMs and despite lower invasiveness, PAM infection with the *S*. Enteritidis ΔSPI1 mutant further significantly increased transcription of these genes (Table 3). Expression of other genes, although sometimes reaching statistical significance, was relatively random. Collectively, these experiments showed that the SPI-1 genes of *S*. *enterica *are involved in the suppression of macrophage proinflammatory signaling.

**Table 3:** Expression of cytokine or immune relevant genes in non-infected PAMs (n.i.) and PAMs 4 and 24 h post infection with the wild-type *S*. Enteritidis and ΔSPI1 mutant.

4 hours post infection with *S*. Enteritidis				24 hours post infection with *S*. Enteritidis		
	**wt**	**ΔSPI1**	**n.i**.	**wt**	**ΔSPI1**	**n.i**.

IL-1β	74.3 ± 59.7	187.5 ± 163.1**	0.62 ± 0.28**	36.8 ± 13.6	238.5 ± 224.5*	0.20 ± 0.19*
IL-8	15.7 ± 3.7	23.4 ± 9.4	0.92 ± 0.57**	5.5 ± 4.7	17.5 ± 6.4	0.19 ± 0.18**
TNFα	57.9 ± 54.9	113.8 ± 105.1**	1.1 ± 1.0**	9.6 ± 7.1	15.6 ± 17.0	0.20 ± 0.07**
IL-23α	7.6 ± 5.3	30.5 ± 22.6**	0.05 ± 0.02**	4.7 ± 2.6	83.1 ± 82.8*	0.014 ± 0.007**
IL-12β	0.025 ± 0.026	0.043 ± 0.038	0.0008 ± 0.0001*	0.29 ± 0.31	3.01 ± 3.63**	0.0009 ± 0.0005**
TGFβ	18.6 ± 6.4	18.7 ± 6.8	11.2 ± 2.1	15.2 ± 7.2	25.4 ± 18.3	12.3 ± 5.6
IL-18	0.56 ± 0.34	0.72 ± 0.39*	0.16 ± 0.08	0.66 ± 0.38	1.13 ± 0.49	0.60 ± 0.58
GM-CSF	0.19 ± 0.15	0.32 ± 0.19*	0.02 ± 0.03**	0.37 ± 0.27	1.92 ± 1.64**	0.015 ± 0.020**
IL-6	0.27 ± 0.37	0.92 ± 1.35	0.0012 ± 0.0011	0.23 ± 0.34	1.79 ± 2.88*	n.d.
NRAMP1	36.8 ± 20.0	32.9 ± 21.1	25.8 ± 12.9*	29.6 ± 17.1	67.9 ± 48.5*	18.0 ± 7.7
SOD1	5.9 ± 2.5	6.3 ± 3.0	4.9 ± 2.3	3.7 ± 1.6	7.5 ± 5.5	3.4 ± 1.4
phox47	4.7 ± 1.4	6.5 ± 1.7*	5.5 ± 1.7	2.9 ± 0.5	4.2 ± 1.1*	4.2 ± 2.0
iNOS	0.012 ± 0.005	0.064 ± 0.035**	0.0015 ± 0.0004**	0.0055 ± 0.0061	0.026 ± 0.039	0.0025 ± 0.0006

n.d. - not detected, expression too low to be detected by RT PCR. * *p *< 0.05, ** *p *< 0.01 when compared to the PAMs infected with the wild-type strain

### Cytokine response of PAMs to Salmonella hilA, sipB and sipA mutants

**Figure 3:**
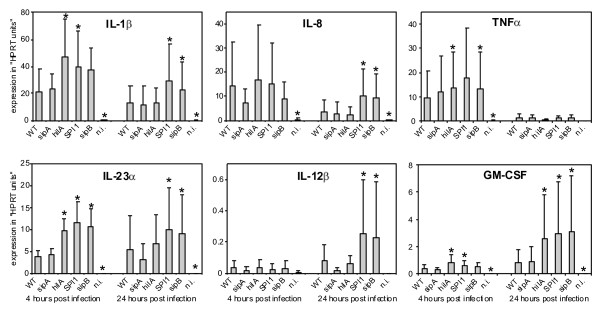
**Cytokine expression of PAMs infected with the wild-type *S*. Enteritidis, *sipA*, *hilA*, ΔSPI1 and *sipB *mutants, and non-infected cells (n.i.)**.

Since Boyen et al. reported that a *hilA *mutant of *S*. Typhimurium stimulated the production of IL-8 by PAMs similarly as did the wild-type strain [15], i.e. different from our observations with the ΔSPI1 mutant, and because Murray and Lee reported on different behavior of ΔSPI1 and *hilA *mutants in mice [2], we tested the cytokine response of PAMs to *hilA*, *sipB *and *sipA *mutants of *S*. Enteritidis in the final experiment. For this experiment we only selected 6 cytokines which responded the most in the previous experiments to *Salmonella *infection. The fully invasive *sipA *mutant never differed in stimulation of the cytokine response from the wild-type *S*. Enteritidis. *hilA *and *sipB *mutants were of reduced invasiveness (Figure 1) and 4 h after the infection, these mutants stimulated cytokine expression of PAMs similar to the ΔSPI1 mutant i.e. higher than did the wild-type *S*. Enteritidis. Interestingly, 24 h post infection, while the *sipB *mutant still behaved similarly to the ΔSPI1 mutant, the *hilA *mutant no longer stimulated the expression of IL-1β, IL-8, IL-12β or IL-23α more than did the wild-type *S*. Enteritidis (Figure 3).

## Discussion

We observed lower invasiveness of ΔSPI1, *hilA *and *sipB *mutants when compared with the wild-type strains or *sipA *mutant, similar to previous reports [15-17]. Also as in other studies, we observed SPI1-dependent macrophage cytotoxicity [17-19], although this was significant only in *S*. Typhimurium and not in *S*. Enteritidis. However, despite the lower invasiveness, we observed higher proinflammatory cytokine expression in PAMs in a response to the infections with ΔSPI1 mutants than to the infections with the wild-type strains. If we calculated the PAM signaling per a single intracellular *Salmonella*, then signaling by a macrophage infected with the wild-type *Salmonella *was nearly 100 times lower than the signaling by the macrophages infected with the ΔSPI1 mutants. PAMs represent a rather specialized macrophage population ready to inactivate pathogens entering the respiratory tract. In their immediate readiness for the pathogen inactivation, they resemble resident macrophages in Peyer's patches of the intestinal tract with which *S*. *enterica *may experience a similar interaction. This is consistent with the results of Monack et al., who demonstrated S. Typhimurium SPI1-dependent macrophage cytotoxicity in Peyer's patches in mice [9] and with the results of Santos et al. who observed apoptotic cells in lymphoid nodules in gut loops of calves infected with *S*. Typhimurium [20]. All of this shows that SPI-1 genes have additional functions besides the invasion of non-professional phagocytes, further supported by recent findings that SPI-1 T3SS-secreted SopB protein is translocated by *Salmonella *inside macrophages [21] or that the SPI-1 T3SS is expressed in *S*. Typhimurium colonizing the liver of mice [22,23].

Our observations were quite different from most of the previous reports on SPI-1 function in *Salmonella *pathogenesis usually stressing the role of SPI-1 T3SS in cell invasion. This could be influenced by the fact that the majority of studies have been performed either with permanent cell lines of epithelial origin or in a gut loop model in which epithelial cells and other cells different from macrophages numerically dominate. In fact, we only found two published experiments in which primary macrophages and non-invasive SPI-1 mutants were used followed by the measurement of cytokine responses. However, Obregon et al. measured only IL-18 expression in human alveolar macrophages by ELISA and RT PCR and similar to our results, they did not find any differences in intracellular amounts of IL-18 in macrophages infected with the wild-type *S*. Typhimurium or non-invasive and non-cytotoxic *sipB *mutant [24]. In the second study, Boyen et al. reported no difference in the secretion of IL-8 into medium of porcine alveolar macrophages 4 h after the infection with wild-type *S*. Typhimurium, *hilA*, *sipA *and *sipB *mutants [15]. The different results could be caused by different protocols used (ELISA vers RT PCR) since our ELISA assay also did not indicate a significant difference, and also by a short exposure of PAMs to *S*. Typhimurium in the study of Boyen et al. because we observed more significant and reproducible differences in the PAM response 24 h rather than at 4 h after the infection with *Salmonella*. However, even in the study of Boyen et al. the same IL-8 production in a response to differently invasive mutants indicated that fewer macrophages infected with the *hilA *and *sipB *mutants must have produced more IL-8 than macrophages invaded by the wild-type *S*. Typhimurium.

Additional experiments with non-invasive *hilA *and *sipB *mutants showed that *S*. Enteritidis internalization was not strictly required for the suppression of macrophage signaling because 24 h post infection, the *hilA *mutant no longer behaved as the ΔSPI1 or *sipB *mutants. Although the explanation for this observation will require additional experiments for understanding it in necessary detail, it may have been caused by the presence of a suppressor protein encoded by SPI-1 as proposed by Murray and Lee [2]. An alternative explanation might be that a low residual expression of SPI-1-encoded T3SS even in the absence of *hilA *limits this mutant in invasion but not in the injection of low but sufficient amounts of *Salmonella *secreted proteins, especially after 24 h of interaction, which then interfere with the cytokine signaling. We therefore concluded that SPI-1-encoded genes are required not only for *Salmonella *cell invasion but also for interference with macrophage cytokine signaling. Moreover, due to the requirement of *sipB *in the modification of macrophage cytokine signaling and its similarity to *ipaB *of *Shigella *sp. and *bipB *of *Burkholderia *sp., this phenomenon might also be common to other bacterial pathogens different from *Salmonella enterica*.

## Competing interests

The authors declare that they have no competing interests.

## Authors' contributions

BP, MC and JV designed and performed RT PCR, PO, HS and JM prepared PAMs, DK constructed the mutants, and MF and IR designed the experiment and wrote the manuscript. All authors read and approved the final manuscript.
